# Rationale for Quality Assurance in Fecal Egg Monitoring of Soil-Transmitted Helminthiasis

**DOI:** 10.4269/ajtmh.15-0463

**Published:** 2016-09-07

**Authors:** David J. L. Hoekendijk, Philip C. Hill, Stephen J. Sowerby

**Affiliations:** ^1^ Centre for International Health, University of Otago, Dunedin, New Zealand; ^2^ Centre for Bioengineering and Nanomedicine, University of Otago, Dunedin, New Zealand; ^3^ Department of Biochemistry, University of Otago, Dunedin, New Zealand

## Abstract

Substantial investment has been made into the once “neglected” tropical disease, soil-transmitted helminthiasis, and into control programs that operate within a framework of mapping baseline disease distribution, measuring the effectiveness of applied interventions, establishing when to cease drug administration, and for posttreatment evaluations. However, critical to each of these stages is the determination of helminth infection. The limitations of traditional microscope-based fecal egg diagnostics have not provided quality assurance in the monitoring of parasite disease and suboptimal treatment regimes provide for the potential development of parasite resistance to anthelmintic drugs. Improved diagnostic and surveillance tools are required to protect therapeutic effectiveness and to maintain funder confidence. Such tools may be on the horizon with emergent technologies that offer potential for enhanced visualization and quality-assured quantitation of helminth eggs.

## Investment Into the Problem of Soil-Transmitted Helminthiasis

Soil-transmitted helminths (STHs) are parasitic nematodes of the gastrointestinal tract of humans and contribute to the group of neglected tropical diseases (NTD). Infection occurs via contact with fecal-contaminated soil in typically tropical and subtropical geographical areas, and is exacerbated by poor sanitation.^1^,^2^ Parasite entry into the host is via penetration of the skin or ingestion of the infective larvae and predominately affects deprived people from low socioeconomic communities of the developing world. STHs are a leading cause of stunted growth and retardation, with significant morbidity in school-aged children, the primary targets of anthelminthic control.^1^,^2^ Various estimates suggest over 1 billion people are infected with one or more STHs, whereas a further 4.5 billion people are at risk, with the most common STHs being roundworm (*Ascaris lumbricoides*), whipworm (*Trichuris trichiura*), and hookworms (*Necator americanus* and *Ancylostoma duodenale*).^1^,^2^ Significant investments from the Bill & Melinda Gates Foundation, the Wellcome Trust, the Uniting to Combat NTD coalition, and a number of governmental and private sector organizations have been committed to the problem of STH. We estimate that approximately US$400 million over the period 2007–2016 (Table 1) has been received or pledged for STH basic science, drug and vaccine research, and STH control programs. A further investment of US$720 million to $1.03 billion will be required for mass drug administration (MDA) until 2020.^3^ It is our perspective that quality-assured STH monitoring is going to be increasingly necessary to provide funder confidence, particularly as any substantial new investment in STH requires the assurance that anthelmintic management will limit the potential for drug resistance.^4^,^5^

## The Need for Quality-Assured STH Monitoring

STH control programs rely on diagnostic methods to determine the baseline infection rate, the effectiveness, and impact of the applied interventions, when to cease drug interventions, and for posttreatment evaluations.^6^ Monitoring gastrointestinal parasites in both human and veterinary health is most often by fecal egg counting (FEC) of stool material by trained technicians capable of sample collection, preparation, optical microscopy, and microscope image interpretation. The Kato-Katz method has been the World Health Organization (WHO) standard for diagnosing STHs in humans for nearly two decades due to its low cost and minimal equipment requirement, but many of the FEC methods developed for the veterinary field also hold promise for human application.^5^–^9^ Veterinary techniques being adapted for human use include the McMaster method, common in agricultural parasitology, and the FLOtation TrAnslation Cringoli (FLOTAC) and Mini-FLOTAC, which have higher sensitivities than Kato-Katz and McMaster.^7^,^8^

A major limitation of traditional fecal egg microscopy is the requirement for a localized “expert” in parasite egg identification at the point of sample analysis to distinguish between a range of different microscopic image features such as pollen, air bubbles, and other confounding debris from different parasite ova, which range in size from 20 to 200 μm. In the Kato-Katz method, a volumetric semisolid stool specimen is spread thinly over an area of approximately 490 mm^2^, which equates to approximately 31 and 197 nonoverlapping fields of view (FOV) at 40× and 100× magnification, respectively.^10^ For the McMaster and FLOTAC methods, the areas of fluidized stool to be imaged range from 100 to 324 mm^2^.^10^ The lack of routine quality assurance in STH monitoring is complicated by the technical challenge of accurately counting helminth eggs.^11^ Stool specimens are inherently unstable and prone to rapid deterioration, which limit their storage and transport for off-site evaluation,^12^ and any putative reevaluation. Kato-Katz preparations of hookworm ova clear rapidly such that it is impractical to store slides and virtually impossible to assess technician error rates.^11^ Egg count errors can have a significant impact on reporting anthelmintic drug efficacy when reports are based on cure rate.^13^ Error is not uncommon in diagnostic microscopy and digital slide recording permits images of samples to be easily reexamined to provide quality assurance.^14^ However, the acquisition of digital microscope images from traditional FEC methods with many FOV cannot easily be achieved and there are impracticalities with recording and storing large numbers of images per sample.

Calls have been made for WHO to review current diagnostic guidelines given the present challenges of FEC.^11^ The need for localized expertise and the lack of quality assurance in FEC have resulted in poor monitoring of STHs.^11^ Diagnostic sensitivity in drug intervention programs needs to be consistent across the program phases and precise quantitative assessment is only required when dealing with low occurrences of low infection intensity.^6^ Molecular techniques applied to STHs reportedly have high specificity and sensitivity, providing putative diagnostic tools for species-specific identification. The polymerase chain reaction has enabled nucleic acid amplification–based identification and quantitation of roundworm, whipworm, and hookworm.^15^ However, the challenges of sample preparation and the presence of enzyme-inhibitory substances in fecal matter require sophisticated laboratory resources and trained personnel, which make the present molecular techniques difficult to deploy in the field. Although FEC only reflects the pattern of egg shedding,^15^ it still remains a practical solution for widespread STH monitoring. The limitations of STH monitoring inevitably exacerbate suboptimal treatment regimes and the potential development of resistance to anthelmintic drugs, as exemplified by localized hookworm resistance in humans^16^ and widespread anthelmintic resistance in sheep and cattle parasites.^17^ Drug resistance in animals reduces the therapeutic tools available to effectively manage helminths.^17^

## Emerging STH Monitoring Technologies

An ideal test for STH would be inexpensive, simple to use, require minimal or no training, and provide quality-assured quantitative diagnosis. Large-scale MDA programs often work in remote areas with little infrastructure,^1^,^2^ and would benefit from field-deployable diagnostic tools to work in parallel with drug administration regimes, within school settings and at the community volunteer level. There is a need to mitigate the logistical challenges and the constraints of collecting, transporting, and storing clinical specimens and associated equipment.^12^

Prototype devices that integrate mobile phone technology with various portable microscopy attachments have demonstrated fecal egg imaging capability.^18^–^20^ The notion of digitizing and transmitting single FOV images could eliminate the need for trained parasitology microscopists from the field and aid in quality-assured monitoring of STHs by providing storable images for reexamination.^10^,^19^ Additional features could inform precise geographical distribution of STH infection via global positioning systems and provide rapid transmission of relevant data to cloud-based services for widespread dissemination and geospatial mapping.^5^ This capability would enable remote access to parasitology expertise and the rapid communication of information to relevant health-care service providers and government subsidiaries. Near real-time technologies have the potential to integrate STH disease surveillance systems with an auditable record for quality-assured diagnosis and potential high specimen throughput.

Two new commercially available helminth parasite monitoring systems have recently emerged from the veterinary field: The FECPAK^G2™^ developed by Techion Group Limited (www.techiongroup.co.nz), is a remote location FEC system enabling quality-assured diagnosis of gastrointestinal nematode infection. In this system, single FOV images, of parasite ova accumulated by an innovative fluidic system are acquired at the site of analysis via a purpose built portable, autonomously operated digital photo microscope and transmitted to “experts” via the Microsoft Azure Cloud system (Microsoft Corp., Redmond, WA). A smartphone version of this technology has been developed.^19^ MEP Equine Solutions LLC (www.theparasightsystem.com) also offer mobile phone-based FEC microscopy for specific parasite ova. Their product, Parasight System^™^, detects fluorescently labeled eggs in the stool of animals.^20^ Originally developed to service agricultural parasitology, these two technology platforms are likely to be useful for human parasite assessment given the transferability of the McMaster and FLOTAC methods, and are adapted to provide quality-assured human parasite monitoring in STH control programs.

## Conclusions

Continued investment into the management of STHs will require the implementation of improved diagnostic and surveillance tools to safeguard therapeutic effectiveness and to provide confidence to the funders of STH research and control programs. Emerging microscope-based fecal egg methods developed for the veterinary sector should be evaluated for human application with the goal to better inform control programs with quality-assured monitoring of STH.

## Figures and Tables

**Table 1:** Funding into STH basic science, drugs and vaccines research, and STH control programs

Sector	Funder	US$
STH coalition*	Uniting To Combat NTD‡	120,000,000‡‡
Philanthropic	Bill & Melinda Gates Foundation§∥	98,498,000§§
	The Wellcome Trust†§	26,264,000§§
	Others†§**	5,574,000§§
Public†	Government organizations§††	118,808,000§§
Private†	Aggregate pharmaceutical and biotechnology companies§	28,182,000§§
	Total	399,660,000∥∥

STH = soil-transmitted helminths; NTD = neglected tropical diseases.

* Initiated in 2014.

† 2007–2014.

‡ http://www.childrenwithoutworms.org/sth-coalition (Accessed March 3, 2016).

§ http://gfinder.policycures.org/PublicSearchTool/ (Accessed March 3, 2016).

∥ http://www.gatesfoundation.org/How-We-Work/Quick-Links/Grants-Database (Accessed March 3, 2016).

** Anonymous donor, Commission for Research Partnership with Developing Countries, FAIRMED–Health for the Poorest, Fondazione Cariplo, Gary K Michelson, Charitable Foundation, Inc., KFPE, Medicor Foundation, SPRIM, Stanley Thomas Johnson Foundation, UBS Optimus Foundation.

†† Argentinian Ministry of Science, Technology and Productive Innovation (MINCYT); Australian Department of Industry; Australian National Health and Medical Research Council (NHMRC); Brazilian Foundation of Research of the State of Para, Fundavao de Amparo a Pesquisa do Estado do Para (FAPESPA); Brazilian Ministry of Health: Department of Science and Technology (DECIT); Broad Institute; Colombian Department for Science, Technology and Innovation (Colciencias); Dutch Ministry of Foreign Affairs–Directorate General of Development Cooperation (DGIS); Engineering and Physical Sciences Research Council (UK); European Commission (including the Directorate-General for Research and Innovation, and the Directorate-General for Development and Cooperation–EuropeAid); Swedish International Development Agency (SIDA); German Federal Ministry of Education and Research (BMBF); German Research Foundation (DFG); Health Research Council of New Zealand (HRC); New York University School of Medicine; US Centers for Disease Control (CDC); Swiss National Science Foundation (SNSF); US National Institutes of Health (NIH); University of California, San Diego; University of Malaya (including the Tropical Infectious Diseases Research and Education Center, TIDREC); UK Medical Research Council (MRC); Texas Children's Hospital; Washington University.

‡‡ Pledged amount.

§§ Expended amount.

∥∥ Provisional total.
